# Effectiveness of warm compresses in reducing the temperature of febrile children: A pilot randomized clinical trial^*^

**DOI:** 10.1590/1980-220X-REEUSP-2022-0168en

**Published:** 2022-10-07

**Authors:** Mariana Vieira de Souza, Danton Matheus de Souza, Elaine Buchhorn Cintra Damião, Soraia Matilde Marques Buchhorn, Lisabelle Mariano Rossato, Marina de Goés Salvetti

**Affiliations:** 1 Universidade de São Paulo, Escola de Enfermagem, Departamento de Enfermagem Materno-Infantil e Psiquiátrica, São Paulo, SP, Brazil.; 2 Universidade Federal de São Paulo, Escola Paulista de Enfermagem, São Paulo, SP, Brazil.; 3 Universidade de São Paulo, Escola de Enfermagem, Departamento de Enfermagem Médico- Cirúrgica, São Paulo, SP, Brazil.

**Keywords:** Fever, Child, Child, Hospitalized, Clinical Trial, Pediatric Nursing, Nursing Care, Fiebre, Niño, Niño Hospitalizado, Ensayo Clínico, Enfermería Pediátrica, Atención de Enfermería, Febre, Criança, Criança Hospitalizada, Ensaio Clínico, Enfermagem Pediátrica, Cuidados de Enfermagem

## Abstract

**Objective:**

To evaluate the effect of the application of a warm compress in association with the prescribed antipyretic drug compared to the effect of the prescribed antipyretic alone, in reducing fever in hospitalized children.

**Method:**

This is a pilot randomized clinical trial performed in pediatric units of a secondary-level hospital. The convenience sample consisted of 33 children with axillary temperature greater than or equal to 37.8°C (100°F), randomized to the control group (antipyretics) or intervention group (antipyretics + warm compresses). Temperature was monitored in both groups for 3 hours and data were collected using standardized instruments, analyzed using Mann Whitney, Fisher’s Exact, Chi-Square, and ANOVA tests.

**Results:**

The control group consisted of 17 children and the intervention group of 16 children. The temperature of all children decreased over time, with progressive attenuation, with a lower final mean in the control group (p=0.035). In the intervention group, irritability and crying were observed in 12.5% of the children.

**Conclusion:**

The application of warm compresses in association with antipyretics was not effective in reducing fever in hospitalized children compared to the use of pharmacological measures alone.

**Clinical trial registration protocol:**

UTN-U1111-1229-1599.

## RESUMO

**Objetivo:**

Avaliar o efeito da aplicação da compressa morna em associação ao antitérmico prescrito em comparação ao efeito isolado do antitérmico prescrito, na redução da febre em crianças hospitalizadas.

**Método:**

Ensaio clínico randomizado do tipo piloto, realizado em unidades pediátricas de um hospital de nível secundário. A amostra, de conveniência, foi composta por 33 crianças com temperatura axilar maior ou igual a 37,8°C, randomizadas para o grupo controle (antitérmicos) ou grupo intervenção (antitérmicos + compressas mornas). A temperatura foi monitorada em ambos os grupos durante 03 horas e os dados foram coletados por meio de instrumentos padronizados e analisados por meio dos testes Mann Whitney, Exato de Fisher, Qui-Quadrado e ANOVA.

**Resultados:**

O grupo controle foi composto por 17 crianças e o grupo intervenção por 16 crianças. A temperatura de todas as crianças diminuiu com o tempo, com atenuação progressiva, com média final menor no grupo controle (p=0,035). No grupo intervenção observou-se irritabilidade e choro em 12,5% das crianças.

**Conclusão:**

A aplicação de compressa morna em associação ao antitérmico não se mostrou eficaz na redução da febre em crianças hospitalizadas em comparação ao uso de medidas farmacológicas isoladas.

## RESUMEN

**Objetivo:**

Evaluar el efecto de la aplicación de compresa tibia en la asociación al antitérmico prescripto en comparación al efecto aislado del antitérmico prescrito, en la reducción de la fiebre en niños hospitalizados.

**Método:**

Ensayo clínico aleatorizado del tipo piloto, realizado en unidades pediátricas de un hospital de nivel secundario. La muestra, de conveniencia, fue compuesta por 33 niños con temperatura axilar superior o igual a 37,8°C, aleatorizados para el grupo control (antitérmicos) o grupo intervención (antitérmicos + compresas tibias). La temperatura fue monitoreada en ambos grupos durante 03 horas y los datos fueron recolectados a través de instrumentos padronizados y analizados por medio de las pruebas Mann Whitney, Exacta de Fisher, Chi-Cuadrada y ANOVA.

**Resultados:**

El grupo control fue compuesto por 17 niños y el grupo intervención por 16 niños. La temperatura de todos los niños bajó con el tiempo, con atenuación progresiva, con promedio final inferior en el grupo control (p=0,035). En el grupo intervención se observó irritabilidad y lloro en el 12,5% de los niños.

**Conclusión:**

La aplicación de compresa tibia en asociación al antitérmico no se mostró eficaz en la reducción de la fiebre en niños hospitalizados en comparación al uso de medidas farmacológicas aisladas.

## INTRODUCTION

Fever is a common clinical entity in children, but despite being self-limited and an adaptive physiological mechanism in isolation, parents tend to perceive the condition as a risk factor and disease severity, which produces fear, anxiety and insecurity and, frequently, leads to seek urgency and emergency services for assistance^(1,2)^, representing approximately 30-40% of attendances in these services^(2,3)^.

In Brazil, in an emergency room, in 2016, there were 407 visits of febrile children^(2)^. This high demand can be explained by the phenomenon of Fever phobia^(4–6)^, in which parents have mistaken beliefs about the febrile condition, which produces exaggerated and irrational fear with a tendency to take hasty measures^(5,7,8)^. An American study with 230 parents observed that 81% took the child with fever to the health service due to the belief that the condition would lead to a seizure (32%), to the child’s death (32%), to brain damage (15%), and the lowering of the level of consciousness (6%) as a result of an infectious condition (3%)^(4)^. The exaggerated search for health services can lead to iatrogenic actions, including unnecessary tests, indiscriminate and inappropriate prescription of antibiotics, antipyretics, analgesics, in addition to nursing care^(2)^.

Fever clinical management is diverse. The use of pharmacological methods, represented by antipyretics, is the first choice^(9–11)^, but non-pharmacological methods such as baths, sponging, compresses, ice packs, refrigerated blankets, fluid intake, removal of clothes, and room ventilation are also widely used^(12)^, both among parents and health professionals^(2,3,6,13)^.

Current investigations, however, demonstrate that many of these practices lack clear evidence for their use^(5,10,12,14)^, as in the case of warm compresses, with controversial use^(15,16)^. In Saudi Arabia, in a cross-sectional investigation with 250 parents, it was observed that 84% of them reported using compresses at home^(17)^. In health services, it is observed that nurses make use of compresses due to their empirical experience in the non-pharmacological management of fever^(18)^, especially for children aged 1 month to 5 years old^(10)^.

The use of compresses has been present in home practice for decades, due to the belief that when the child’s skin is externally cooled, its temperature tends to decrease^(13,19)^; however, there have been no studies yet, national or international, which clearly demonstrate whether this hypothesis is true. What is known is that, when exposing the child to warm water, vasoconstriction and tremors can be triggered, which consequently influences fever^(12)^.

In clinical practice, many nursing professionals translate popular practice into care^(5)^, with the use of a warm compress as a non-pharmacological measure; however, this empirical performance is worrisome, since nurses and their team have the social representation of “figures of knowledge”, and the population, when observing the use of this technique by professionals, tends to repeat it at home, as seen in the studies mentioned^(12–14)^. The dissemination of a practice that does not have its effectiveness proven in the international literature is a phenomenon of potential investigation.

Based on pre-existing literature which portrays that the use of cold^(12,19)^ and hot^(20)^ water is not effective in reducing fever, but with differences regarding the use of warm water^(11,19)^, this study considered the warm compress an object of research. When observing that the literature portrays possible adverse effects of the use of warm water^(12)^, the null hypothesis was supported that the administration of antipyretic alone is as effective in reducing the mean body temperature of febrile children as the reduction in temperature that occurred with children who received antipyretic associated with the intervention with warm compresses.

Thus, the following concern emerged: “What is the effectiveness of the use of warm compresses associated with and compared to antipyretics in reducing the temperature of febrile children?”, and this study aimed to evaluate the effect of the application of warm compresses in association with the prescribed antipyretic compared to the isolated effect of the prescribed antipyretic, in reducing fever in hospitalized children.

## METHOD

### Design of Study

This is a pilot randomized clinical trial, which tested the application of warm compresses in febrile children associated with the administration of antipyretics compared to the administration of the drug alone to control fever. To guide the description of this study findings, the recommendations of the instrument Consolidated Standards of Reporting Trials (CONSORT) were followed^(21)^.

### Local

The investigation was carried out at the University Hospital of the Universidade de São Paulo (HU-USP), a secondary-level teaching hospital, with characteristics of a tertiary hospital, located in the city of São Paulo-SP, Brazil. Data collection was performed in a pediatric department, in pediatric inpatient units (PIU), pediatric intensive care units (PICU), and children’s emergency room (PER), from June 2019 to January 2020.

### Population and Selection Criteria

The population of this study consisted of febrile children. The convenience sample included 33 children, based on the following criteria: age group between 1 month and 11 years, 11 months, and 29 days; axillary temperature greater than or equal to 37.8°C (100°F), and with an interval between the antipyretic administration and the beginning of application of the intervention studied of up to 10 minutes. Children diagnosed with malignant hyperthermia, neurological dysfunction, and those undergoing any procedure during the period of up to 3 hours after antipyretic administration were excluded. It is worth mentioning that the inclusion of the same child was allowed, if his/her last inclusion in the study had occurred in a time greater than 12 hours and he/she was not under the effect of antipyretic, establishing a minimum interval of 06 hours from the last antipyretic used.

### Sample Definition

The sample adopted was of convenience, selecting children who were in the pediatric sectors and had fever during the period of data collection. The sample size was defined by a statistician, based on previous studies with similar methodologies^(8,12)^, with a total of 288 children; however, in the pre-established period, only 37 were collected, which makes this study a pilot randomized clinical trial.

### Data Collection

Prior to collection, randomization was performed through a website (randomizer.org), which allowed the creation of numbered envelopes in sequential order. Data collection was performed by researchers, nurses, and previously trained nursing residents working in the co-participating institution. There was no blinding of the participating child and family nor of the evaluating professional, considering that the warm compress is an intervention visible to all. When including a child in the study, the envelope corresponding to the participation number was opened, indicating which group they would randomly participate in: 1) Control group (CG): children with fever undergoing pharmacological treatment, and 2) Intervention Group (IG): children with fever undergoing pharmacological treatment associated with the application of warm compresses.

The main researcher created a field manual with the study variables and the procedures to be followed by the researchers/collectors. A fever protocol was also created, attached to the children’s medical records, to standardize and inform the teams of the different shifts about the study participants.

The children’s guardians were invited to participate by the main researcher and/or collector, with clarification on the purpose of the study and data collection, with the joint reading of the Free and Informed Consent Form (FICF) and, after agreement to participate, signing of the document in two copies.

For the study operationalization, the child in the CG received drug therapy, previously prescribed by the medical team, and was kept dressed, carrying out his/her activities. The child in the IG received drug therapy and within 10 minutes after administration, to avoid interference from therapy in the intervention, warm compresses were applied, with water between 34-37°C (93.2°F-98.6°F), in the frontal, axillary and inguinal regions, for 15 minutes, period in which the child was kept undressed, with no coverings; then, the child’s body was dried. For the compresses, a multipurpose cloth Wiper Pro50^®^ size 25 x 28 cm was used; Termomed^®^ digital clinical thermometer for axillary temperature measurement; and digital thermometer with inside and outside maximum and minimum temperature to measure the water temperature. The axillary temperature with the electronic thermometer was chosen due to the ease of measurement, and because it was a comfortable method, already known by children^(22)^.

The temperature measurement in both groups took place at the following times: M0- Diagnosis of fever by the hospital employee; M1- Confirmation of fever by the researcher with the standardized digital thermometer, with the inclusion of the child in the study (Time 0-T0); M2- Checking the child’s temperature 30 minutes after the antipyretic administration (T1); M3- Checking the child’s temperature 60 minutes after the antipyretic administration (T2), and M4- Checking the child’s temperature 3 hours after the antipyretic administration (T3). To measure the child’s axillary temperature and the temperature of the water during the intervention, thermometers standardized by the researcher were used.

During data collection, instruments were filled out with the following variables: 1) Primary variable: reduction in axillary temperature, in degrees Celsius, using the digital thermometer, and 2) Secondary variables: age; sex; inpatient unit; length of stay; medical diagnostic; medications in use; irritability, tremors and child cry.

### Data Analysis and Treatment

Data were organized in a database using Microsoft Office Excel 2007^®^, Statistica version 13.5.0.17^®^, and R software (packages NLME^®^, IME4^®^, HLM Diag^®^). Descriptive data analysis was performed by simple frequency distribution, measure of central tendency (mean), and dispersion measures (variance and standard deviation) according to the studied variable categorization. To analyze the association of variables, the following statistical tests were used: Mann Whitney, Fisher’s Exact, Chi-Square and Analysis of Variance (ANOVA). A statistical significance level of 5% (p<0.05) was adopted.

### ETHICAL ASPECTS

The study received ethical approval from the Research Ethics Committee (*CEP*) of the Nursing School of Universidade de São Paulo (CAAE No. 06472819.8.0000.5392; Opinion No. 3.574.282) and from the *CEP* of the University Hospital of Universidade de São Paulo (CAAE No. 06472818.8.3001.0076; Opinion No. 3.604.872). The ethical precepts of Resolution No. 466/12 of the National Health Council were followed. The research was registered in the Clinical Trials Registry (*ReBEC*) under the protocol number UTN-U1111-1229-1599, but this number was generated months after the project was sent, when data collection was already being carried out.

## RESULTS

**Figure 1.**
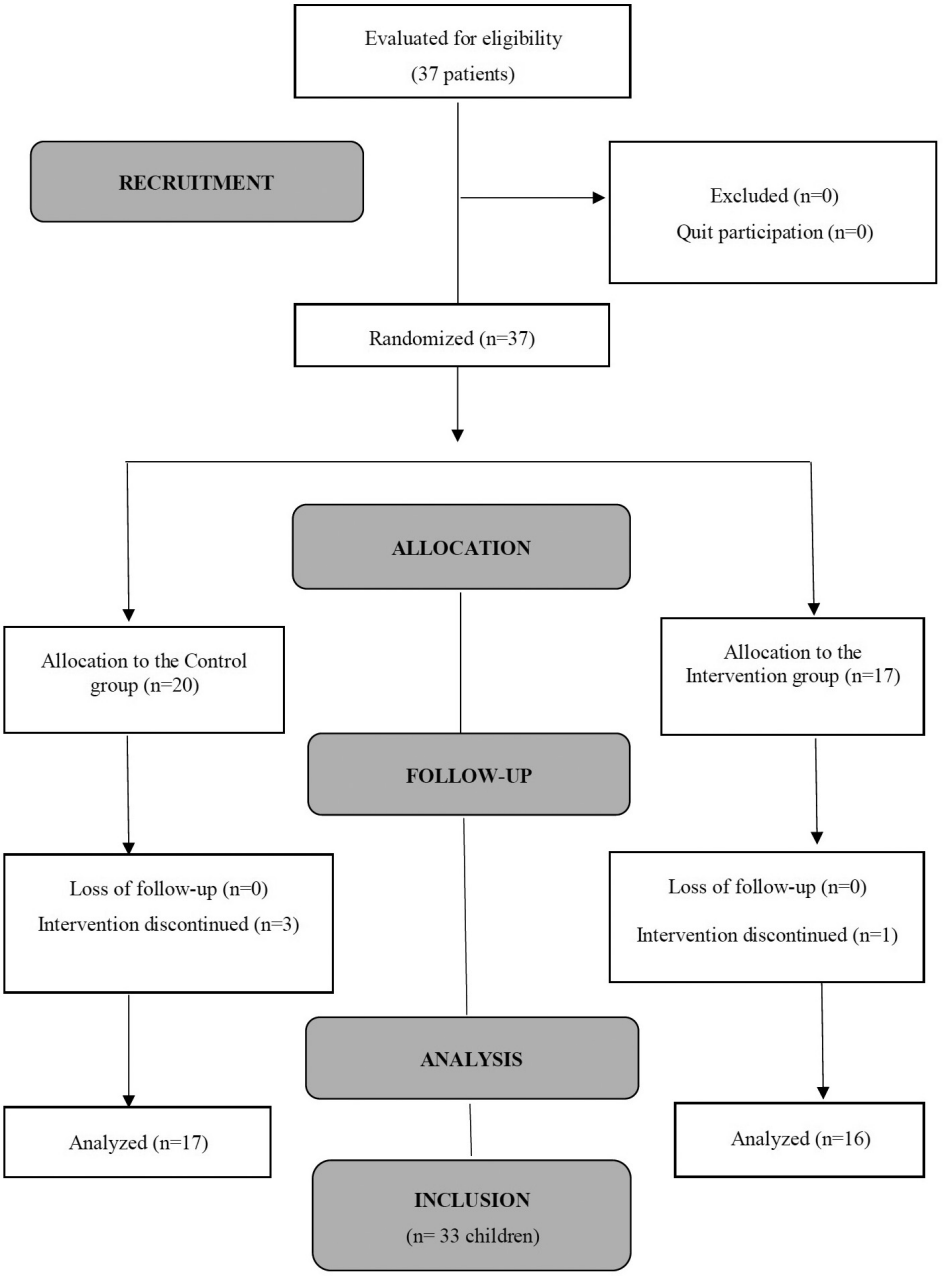
Flowchart of children distribution. São Paulo-SP, Brazil. 2020.

The initial sample consisted of 37 children, 10 from the PER, 15 from the PIU and 12 from the PICU. Four children were excluded due to failures to fill in the collection instruments. Thus, the final sample consisted of 33 children, 17 in the CG and 16 in the IG (Figure 1).

There was no significant difference between the groups in terms of age, sex, hospitalization unit, time taken to participate in the research, medication used, and pathology (Table 1).

**Table 1.** Characterization of children according to groups - São Paulo, SP, Brazil, 2020.

Variables	CG (N=17)		IG (N=16)		p-Value
	Mean	SD^1^	Mean	SD^1^	
Age					0.125****
Months	37	31	23	26
	**N**	**%**	**N**	**%**	
Sex
Female	9	53%	7	44%	0.597**
Male	8	47%	9	56%
Place of admission
PER	6	35%	1	6%	0.112***
PIU	7	41%	8	50%
PICU	4	24%	7	44%
Inclusion in the research (hours)*
≤ 24 hours	10	59%	3	19%	0.052***
24–72 hours	4	23%	9	56%
72 hours	3	18%	4	25%
Medication
Paracetamol	2	12%	4	25%	0.654***
Dipyrone	14	88%	12	75%
Diagnosis
Respiratory pathologies	12	70%	9	56%	0.728***
Infectious pathologies	2	12%	3	19%
Various pathologies	1	6%	2	12.5%
Gastrointestinal pathologies	1	6%	2	12.5%
Unidentified pathologies	1	6%	–	–

Notes: ^1^Standard deviation; *Number of days of hospitalization before the child’s inclusion in the research; **Chi-square test; ***Fisher’s exact test; ****Mann Whitney Test.

Data analysis showed that all children’s temperatures decreased over time, with progressive attenuation, regardless of allocation group; however, at the end, the CG presented a lower final mean temperature (p=0.035) (Table 2). It was found, however, that 12.5% of the children in the IG presented irritability and crying, a fact that was not observed in the CG.

**Table 2.** Comparison of temperatures according to measurement times and allocation group - São Paulo, SP, Brazil, 2020.

Time (minutes)	Group	N	Sum	Mean	Standard deviation	p- Value
**T0 (antipyretic)**	CG	17	653.5	38.4	± 0.6	0.526*
	IG	16	612.1	38.3	± 0.3	
**T1 (30 minutes)**	CG	17	644.8	37.9	± 0.6	0.505**
	IG	16	604.6	37.8	± 0.6	
**T2 (60 minutes)**	CG	17	634.2	37.3	± 0.5	0.594**
	IG	16	598.5	37.4	± 0.6	
**T3 (180 minutes)**	CG	17	622.1	36	± 0.6	0.035**
	IG	16	593.5	37.1	± 0.7	

Notes: *Mann-Whitney test; **Student t test.

**Figure 2.**
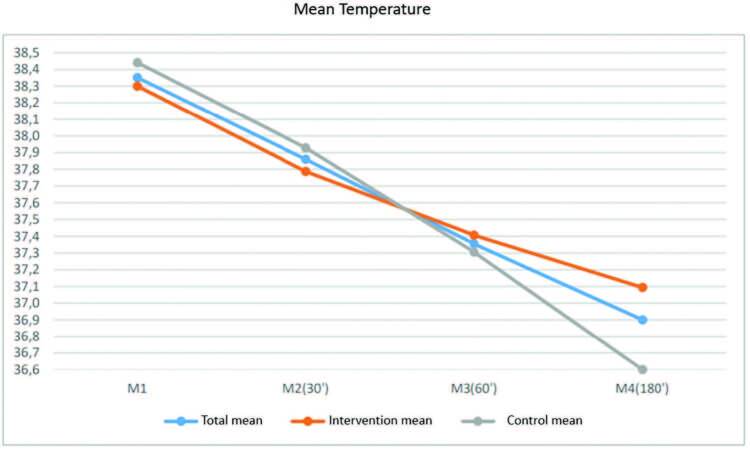
Variation of the mean temperature of the control and intervention groups and the total mean according to the moments of data collection. São Paulo-SP, Brazil. 2020.

**Figure 3.**
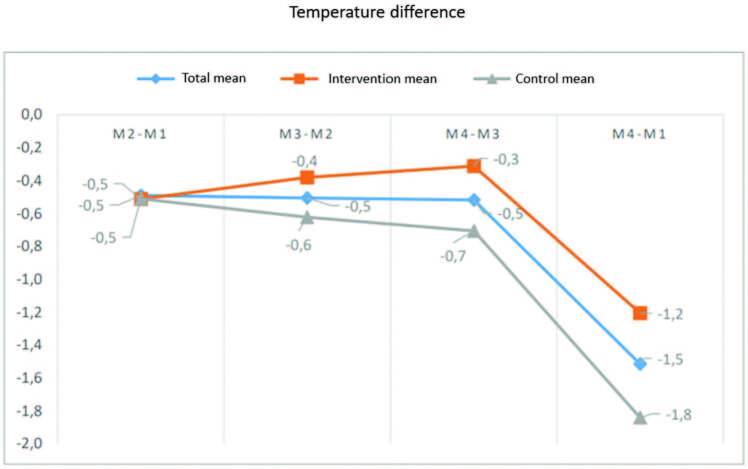
Comparison of the temperature difference between the two groups according to the moment of the study. São Paulo-SP, Brazil. 2020.

In M4, only 12.1% of the children remained feverish, with mean temperature of 37.9°C (100.22°F); 21.2% became subfebrile (37.1-37.7°C [98.78°F-99.86°F]), and 66.7% were afebrile. The comparison of the mean temperature in the two groups over time showed a greater drop in temperature at the beginning of the application of warm compresses in the IG and a lower mean final temperature in the CG. Both groups showed a constant decreasing line of temperature drop, with no significant difference between the groups (Figures2 and 3).

The ANOVA test was used to analyze repeated data and there was no difference in temperature regarding the inpatient unit or the child’s allocation group in the study (IG or CG).

## DISCUSSION

There are different understandings regarding the management of fever, especially in relation to non-pharmacological measures, with conceptual disparity related to the effectiveness of different methods, which are used both at home, by parents, and in the hospital service^(2,11)^. In the context of health care, nurses play a unique role in the care of febrile children and often make use of non-pharmacological measures based on individual convictions and clinical experience, with no scientific evidence^(2,12)^.

This scenario is evidenced in the literature, as it is observed that professionals’ actions are based on personal beliefs, clinical experience, local care protocols, history of care for children with febrile seizures, and parental requests^(23,24)^. Low scientific empowerment can result in ineffective care, since it does not bring benefits to the child, or even iatrogenic care, considering the discomfort caused by the use of physical methods with low accuracy^(2,6,9)^.

The use of warm compresses is an empirical practice widely used by nurses in the management of hospitalized febrile children^(10)^. However, to date, there is no consensus on the effectiveness of the method, with few publications and divergent results^(12)^. Some studies have shown that the warm compress intervention has a synergistic effect with the antipyretic, being faster and more lasting in reducing the body temperature of the febrile child than the administration of the antipyretic alone. However, other studies have shown that these interventions do not make any difference, with the medication being solely responsible for the drop in the child’s temperature^(12,14,15)^. In this study, it was observed that, at the end of the intervention, the CG had a lower final mean temperature compared to the warm compress group.

In an integrative literature review^(14)^, carried out in Brazil, with 16 studies published between 1985 and 2011, only four studies showed moderate evidence and recommendation for warm compresses. However, it should be noted that the aforementioned studies were carried out more than 20 years ago, and one of them recommended the use of cold compresses, with a low level of evidence.

The use of warm compresses was also tested in the adult population. In a randomized clinical trial carried out in Brazil, in which the use of warm compresses or ice packs associated with antipyretics was compared, the results showed no significant difference regarding the use of warm compresses or ice packs compared to antipyretics^(15)^, similar to the findings of the present study, which differs because it was performed in the pediatric population

There are disagreements in the literature regarding the value of body temperature for fever. In pediatric clinical practice, fever in children is considered to be body temperature ≥37.8°C (100°F) in most cases. However, national and international studies report as eligibility criteria children with body temperature above 38°C (100.4°F) or even above 38.3°C (100.94°F)^(10,14,16)^. Although this study started from a lower temperature, at the end of three hours, children’s mean body temperature was mostly below 37ºC (98.6°F), and the CG had a significantly lower mean temperature (p=0.035).

However, it should be noted that the drop in temperature in the IG, at all times, was lower than the drop observed in the CG. It was expected that the IG would have a greater decrease in temperature, compared to the CG, if the intervention was relevant. Another point is that the child in the IG was undressed during the 15 minutes of intervention, and as shown in the literature, the removal of excess clothing is also a relevant non-pharmacological measure^(11,12)^; however, in the end, the CG showed a greater mean reduction in temperature, and it can be inferred that the intervention of warm compresses was not effective in helping to lower the temperature of febrile children compared to the CG, corroborating the aforementioned studies^(14,15)^ and confirming the null hypothesis.

Also, in the integrative review of the literature, carried out in Brazil, it was observed that studies of warm compresses recommend that their use be carried out in situations of high body temperature, between 38.9°C (102.02°F) and 40.6°C (105.08°F), for promoting its rapid reduction, and its use in the care of fever unresponsive to antipyretics^(14)^. Since hyperthermia is the increase in body temperature, resulting from the imbalance between heat production and dissipation, considered in values above 40°C (104°F) and with no response to antipyretics^(25)^, previously mentioned criteria^(14)^, it is worth reflecting: what is the effectiveness of the use of warm compresses in cases of hyperthermia? This is a relevant research question for further studies.

The level of discomfort observed in the survey was small. Only two children (12.5%) in the IG showed irritability (p=0.215) and crying (p=0.769). It is hypothesized that due to this study’s small sample size, or perhaps because Brazil is a tropical country and data were collected during the summer period, a high incidence of discomfort was not observed in the children in the study. Moreover, in practice, the difficulty in carrying out the intervention was observed due to the characteristic behavior of children under 3 years of age, especially in the local PIU where children are often not prostrate.

Physical methods decrease body temperature by increasing the gradient between body temperature and the environment, promoting heat loss mechanisms^(19,25)^, which lead to body compensatory measures with peripheral vasoconstriction, tremors that can generate even more heat^(14)^, increased metabolic demand, marginal cerebrovascular and cardiac supply^(26)^, and rebound hypothermia, which should also be the focus of attention of the nursing team^(12,15)^. These situations of discomfort were reported in other studies using warm compresses^(10,16,26)^.

The use of warm compresses provides heat loss through conduction and convection mechanisms, similar to the sponging intervention, with loss by conduction, convection, and evaporation^(20,27)^. While there is a lack of research in the national territory addressing the use of warm compresses, there is a high number of international publications on sponging^(11,20,27)^. Although many authors in the national literature consider the term sponging as analogous to the warm compress, there are differences, since in sponging the body, from neck to toe, is gently rubbed with the warm water compress, on the lower and upper limbs^(12)^.

In the international literature there are also divergences regarding the sponging technique^(27)^. In a Brazilian randomized clinical trial with 120 children undergoing associated sponging, and compared to the use of antipyretic alone, it was observed that in the first 15 minutes there was a greater reduction in temperature in the IG (p.<0.001), but from 30 minutes to 120 minutes the reduction was greater in the CG. Crying (52%), irritability (36%) and tremors (1.6%) were observed in the children in the IG and none in the CG^(10)^. Discomfort was also observed in other studies, and a systematic review concluded that the side effects and the short-term effect of the intervention outweigh the clinical benefits of lowering the temperature, not recommending the use of the method^(24)^.

In another randomized clinical trial, carried out in India, with two experimental groups, IG1 (warm sponging) and IG2 (hot sponging), it is observed that the use of hot water can reduce the discomfort provided by the intervention^(26)^. However, in a quasi-experimental study carried out with 20 children with typhoid fever, in which 10 used hot compresses and 10 used warm sponging, without medication, both groups had a significant reduction in body temperature in the first 15 minutes (p.<0.05), but only sponging showed results after 30 minutes (p<0.05), and at the end of the evaluation, after 60 minutes, both were not significant, with an increase in temperature and fever recurrence^(20)^. Thus, the use of hot water is also controversial.

In the present study, it was noted that, although the children in the IG presented a greater drop in temperature at the beginning of the application of warm compresses, the CG had a constant drop in temperature and, after three hours, the mean temperature was 36.6°C (97.88°F), while the IG mean was 37.1°C (98.78°F). This finding corroborates other randomized clinical trials, either on the use of warm compresses^(12,14,15)^ or sponging^(10,16,26)^. This finding can be explained by the mechanism of action of the medications. If administered orally, antipyretics have an action starting after 30 minutes, on average, with plasma peak in 02 hours^(24,26)^. The studies do not provide data regarding the route of medication administration, being only a hypothesis that the reduction in temperature may be related to the use of antipyretics.

Based on an integrative review^(12)^ and systematic reviews^(11,24)^, without meta-analysis, it can be said that the non-pharmacological measures that are still recommended are: stimulation of water intake, removal of excess clothes and blankets, and room ventilation, as long as it does not cause tremors. These measures, in addition to the objective of helping to reduce the temperature, aim to reduce children’s discomfort and improve their general well-being. It is important for the professional to assess the potential benefit and risk of each measure at the time of its prescription and implementation.

One of the problems observed in the use of non- pharmacological measures in the management of fever is related to the difficulty in translating the best scientific evidence, demonstrated by the aforementioned reviews and clinical trials, into professional practice and, consequently, home practice. In this sense, it is necessary to invest in the continuing education of nurses so that the care provided is grounded and qualified, and in the formulation of institutional protocols for fever management, so that knowledge is universally integrated within the service^(12)^, avoiding the misuse of time, both by the nurse who prescribes care and by the professional of the technical nursing team who performs it. It is extremely important that nurses use the best scientific evidence in their work and transfer the knowledge produced to their practice, with qualified guidelines and greater effectiveness in interventions.

This study presented strengths and limitations, which should be highlighted. Among the strengths is the study design, a randomized clinical trial, carried out with a pediatric population, which tested the effects of a non-pharmacological intervention frequently used in clinical practice, but still without clear evidence on its effects. Regarding limitations, the small sample size and the low hospital occupancy rate during the data collection period are highlighted, as well as the data being collected in only one institution, which makes it difficult to generalize findings.

## CONCLUSION

The beneficial effects of the application of warm compresses associated with antipyretics could not be verified in the control of fever in pediatric patients when compared to the use of antipyretic alone. In the group that received warm compresses, irritability and crying were observed in 12.5% of the children. However, the development of new investigations, with larger samples and in different settings, is suggested to confirm these findings. Hopefully, this investigation will contribute to nurses’ decision making regarding the use of warm compresses in febrile children.

## References

[1] El-Radhy AS (2008). Why is the evidence not affecting the practice of fever management?. Arch Dis Child..

[2] Pitoli PJ, Duarte BK, Fragoso AA, Damaceno DG, Marin MJS (2021). Fever in children: parents’ search for urgent and emergency services. Cien Saude Colet..

[3] Sá ACMGN, Silva RM, Capanema FD, Gonçalves LAO, Rocha RL (2018). Childhood fever and its management by parents: a qualitative analysis. Rev Bras Cienc Saude..

[4] Poirier M, Collins EP, McGuire E (2010). Fever phobia: a survey of caregivers of children seen in a pediatric emergency department. Clin Pediatr..

[5] Clericetti CM, Milani GP, Bianchetti MG, Simonetti GD, Fossali EF, Balestra AM (2019). Systematic review finds that fever phobia is a worldwide issue among caregivers and healthcare providers. Acta Paediatr..

[6] Alqudah M, Cowin L, George A, Johnson M (2019). Child fever management: a comparative study of Australian parents with limited and functional health literacy. Nurs Health Sci..

[7] Olympia RP (2016). School nurses on the front lines of medicine: a student with fever and sore throat. NASN Sch Nurse..

[8] Martins M, Abecasis F (2016). Healthcare professionals approach paediatric fever in significantly different ways and fever phobia is not just limited to parents. Acta Paediatr..

[9] Pereira GL, Tavares NU, Mengue SS, Pizzol TS (2013). Therapeutic procedures and use of alternating antipyretic drugs for fever management in children. J Pediatr..

[10] Alves JGB, Almeida NDCM, Almeida CDCM (2008). Tepid sponging plus dipyrone versus dipyrone alone for reducing body temperature in febrile childre. Sao Paulo Med J..

[11] Green C, Krafft H, Guyatt G, Martin D (2021). Symptomatic fever management in children: a systematic review of national and international guidelines. PLoS One..

[12] Souza MV, Damião EBC, Buchhorn SMM, Rossato LM (2021). Non-pharmacological fever and hyperthermia management in children: an integrative review. Acta Paul Enferm.

[13] Villarejo-Rodríguez MG, Rodríguez-Martín B (2019). Parental approach to the management of childhood fever: differences between health professional and non-health professional parents. Int J Environ Res Public Health..

[14] Salgado PO, Silva LCR, Silva PMA, Paiva IRA, Macieira TGR, Chianca TCM (2015). Nursing care to pacient with high body temperature: an integrative review. Rev Min Enferm..

[15] Salgado PO, Silva LCR, Silva PMA, Chianca TCM (2016). Physical methods for the treatment of fever in critically ill patients: a randomized controlled trial.. Rev Esc Enferm USP.

[16] Thomas S, Vijaykumar C, Naik R, Moses PD, Antonisamy B (2009). Comparative effectiveness of tepid sponging and antipyretic drug versus only antipyretic drug in the management of fever among children: a randomized controlled trial. Indian Pediatr..

[17] AlAteeq MM, AlBader BO, Al-Howti SY, Alsharyoufi M, Abdullah JB (2018). Parent’s knowledge and practice in home management of fever in their children in Riyadh, Saudi Arabia. J Family Med Prim Care..

[18] McDougall P, Harrison M (2014). Fever and feverish illness in children under five years. Nurs Stand..

[19] Raak C, Scharbrodt W, Berger B, Boehm K, Martin D (2022). The use of calf compresses for gentle fever reduction. What do we know? A scoping review. Collegian..

[20] Karra AKD, Anas MA, Hafid MA, Rahim R (2019). The difference between the conventional warm compress and tepid sponge technique warm compress in the body temperature changes of pediatric patients with typhoid fever. Jurnal Ners..

[21] Schulz KF, Altman DG, Moher D, CONSORT Group (2010). CONSORT 2010 statement: updated guidelines for reporting parallel group randomized trials. Obstet Gynecol..

[22] El-Radhi AS (2014). Determining fever in children: the search for an ideal thermometer. Br J Nurs..

[23] Camargo FC, Iwamoto HH, Galvão CM, Pereira GA, Andrade RB, Masso GC (2018). Competences and barriers for the evidence-based practice in nursing: an integrative review. Rev Bras Enferm..

[24] Watts R, Robertson J (2012). Non-pharmacological management of fever in otherwise healthy children. JBI Library Syst Rev..

[25] Beard RM, Day MW (2008). Fever and hyperthermia: learn to beat the heat. Nursing..

[26] Pavithra C (2018). Effect of tepid vs warm sponging on body temperature and comfort among children with Pyrexia at Sri Ramakrishna hospital, Coimbatore. IJSAR.

[27] Karin YA, Arsyad NA, Ningsih JF (2021). Tepid sponging and compress plaster on toddlers who have a fever. Jurnal Kebidanan Malahayati..

